# Dry Film Resist Laminated Microfluidic System for Electrical Impedance Measurements

**DOI:** 10.3390/mi12060632

**Published:** 2021-05-29

**Authors:** Yuan Cao, Julia Floehr, Sven Ingebrandt, Uwe Schnakenberg

**Affiliations:** 1 Institute of Materials in Electrical Engineering 1, RWTH Aachen University, Sommerfeldstraße 24, 52074 Aachen, Germany; yuan.cao@iwe1.rwth-aachen.de (Y.C.); ingebrandt@iwe1.rwth-aachen.de (S.I.); 2 Helmholtz-Institute for Biomedical Engineering, RWTH Aachen University Hospital, Pauwelsstraße 30, 52074 Aachen, Germany; jfloehr@ukaachen.de

**Keywords:** dry film resist, SU-8, SUEX, electrical impedance spectroscopy, microelectrode, microfluidic, MEMS, electrical equivalent circuit, hydrodynamic trap

## Abstract

In micro-electrical-mechanical systems (MEMS), thick structures with high aspect ratios are often required. Dry film photoresist (DFR) in various thicknesses can be easily laminated and patterned using standard UV lithography. Here, we present a three-level DFR lamination process of SUEX for a microfluidic chip with embedded, vertically arranged microelectrodes for electrical impedance measurements. To trap and fix the object under test to the electrodes, an aperture is formed in the center of the ring-shaped electrodes in combination with a microfluidic suction channel underneath. In a proof-of-concept, the setup is characterized by electrical impedance measurements with polystyrene and ZrO_2_ spheres. The electrical impedance is most sensitive at approximately 2 kHz, and its magnitudes reveal around 200% higher values when a sphere is trapped. The magnitude values depend on the sizes of the spheres. Electrical equivalent circuits are applied to simulate the experimental results with a close match.

## 1. Introduction

Advanced technologies are used for the fabrication of micro-electro-mechanical systems (MEMS). For many applications, thick structures with high aspect ratios are needed. The processing of liquid photoresists for the fabrication of complex, three-dimensional, multilayered resist structures is challenging and remains cumbersome because of resist deposition over thick pre-existing structured layers. In addition, free-standing structures of considerable heights cannot easily be coated with liquid photoresist to achieve homogeneous layer thicknesses.

To overcome these drawbacks, dry film photoresist (DFR), originally developed for printed circuit boards (PCB), can easily be laminated, also over already structured layers. They show advantages over liquid photoresist processing with regard to simple processing, short processing times, rapid prototyping capabilities, no liquid handling, good conformity, excellent adhesion properties to other substrates as well as no formation of edge beads. Processing is not restricted to silicon or glass wafers but can be carried out on any format of substrates. Furthermore, bridge-type structures or channel covering can be obtained with low mechanical stress inside the DFR layers. It is worthwhile to note that commercially available DFRs exhibit extremely uniform thicknesses, which cannot be achieved by processing liquid resists. By using DFR, the wastage of resist is significantly reduced when compared to liquid resist processing. For thicker DFR structures, lamination of several layers can be carried out. Because we are interested in multi-level lamination processing, we summarize here approaches that already use commercially available DFRs.

One of the first DFRs, called Riston^®^, was introduced in 1970 by DuPont (Wilmington, DE, USA) for applications in the printed circuit board industry [1]. Khalkhal et al. developed a five-level lamination process for the fabrication of molds using 100 µm thick Riston^®^ DFR sheets. Lorenz et al. showed in a four-level lamination process that Riston^®^ DFR is suitable as a mold for electroplating processes when metallic gear and piston sets with upper and lower axle posts need to be manufactured [2]. Here, for the first time, seed layer deposition and subsequent electroplating were carried out in intermediate DRF layers to obtain larger structures over smaller ones. Heuschkel et al. showed that buried microfluidic channels can be fabricated by a two-level Riston^®^ DFR lamination process [3].

Kukharenka et al. used two layers of at least 100 µm thick Ordyl series DFR (Elga Europe, Milano, Italy) in combination with electroplating processes for multilayer electroplated parts [4]. These acrylate-based negative DFRs contain esters with epoxy groups and therefore show excellent adhesion to themselves and to most other substrates as well as chemical stability [5]. However, an aspect ratio of only 2.7:1 was reported [6,7]. Vulto et al. combined four-layer Ordyl DRF lamination for the fabrication of microfluidic channels with microelectrodes electroplated at the bottom substrate [5], whereas Mulloni et al. fabricated a microfluidic device with three-level DFR and electrodes at the top and bottom plate [8]. Trantidou et al. [9] as well as Guijt et al. [10] developed a multilayer lamination process for simple microfluidic channels.

As the first company, IBM developed a liquid epoxy-based negative photoresist based on SU-8, which can be spin-coated in layers of a few hundreds of microns and patterned with conventional UV lithography [11,12]. Compared to the acrylate-based Ordyl DFRs, SU-8 based DFRs exhibit higher resolution and chemical stability but are more expensive and less hydrophilic. Meanwhile, several SU-8-based DFRs are commercially available.

To the best of our knowledge, Abgrall et al. were the first who reported a three-level lamination process of SU-8 DFR. The DFR was laminated as a cover lid for a microfluidic channel to a cross-linked SU-8 layer, which was processed with a liquid resist [13].

SU-8-based PerMX DFR (DuPont) was used by Meier et al. for a six-level process for the fabrication of complex microfluidic networks in combination with laser micromachining [14]. Ajada and Asthana showed that a three-level PerMX DRF film can be successfully used as a mask for deep reactive ion etching of silicon with O_2_/SF_6_/CHF_3_ plasma [15].

TMMF S series (Tokyo Okha Kogyo, Kawasaki, Japan) DFR was used by Wangler et al. for the fabrication of simple microfluidic structures [9]. They report an extremely detailed study on resist processing. An aspect ratio of 9:1 for channel structures and 7:1 for free-standing structures and sealing of wide channel structures up to 2 mm without sagging of the lid layer were obtained with TMMF DFRs. Kalkandjiev et al. reported a two-level lamination process of TMMF DFR in combination with bonding to silicon [16].

DF-1050 (Engineered Materials Systems, Delaware, OH, USA) is 10 times cheaper than TMMF S2000 and exhibits faster processing times, as Courson et al. reported [17]. They developed a five-level lamination process for microfluidic mixer devices, whereas a two-fold lamination process was used for the fabrication of micro-optical-electrical-mechanical system (MOEMS) [18]. Here, thin-film processing of metal films on top of the last DFR layer was carried out to define actuation electrodes.

With the XP SU-8-3000 film series (Nippon Kayaku, Tokyo, Japan), Onoshima et al. fabricated molds for poly(dimethylsiloxane) (PDMS) molding applications [19], whereas, with 35 µm thick Etertec HQ-6100 sheets, Stephan et al. fabricated a microfluidic gradient generator with a total channel thickness of 140 µm with a four-level lamination process [20].

SUEX DFRs were developed by Donald Johnson (DJ MicroLaminates, Sudbury, MA, USA). He introduced the properties of this type of DFR with a three-level lamination process for microfluidic applications [21,22]. Recently, Xie et al. showed the fabrication of LEGO^®^-like bricks, where 500 µm and 200 µm thick SUEX DFRs were laminated and photolithographically patterned, serving as the master for the bricks [23]. Levy et al. laminated up to 10 layers of SUEX DFR and combined a stereolithography-based method for structural material produced with laser-induced forward transfer printing of conductive and resistive elements [24].

Besides commercially available DFRs, micro resist technology GmbH (Berlin, Germany) developed an SU-8-based DFR. In a joint collaboration, also with the Fraunhofer Research Institution for Microsystems and Solid State Technologies (Munich, Germany), we developed a six-level lamination process of 33 µm thick DFR layers for the fabrication of 3D microfluidic devices [25].

With regard to the carrying out of the lamination process, the presented literature survey reveals that the first DFR layer is typically laminated onto the substrate at higher pressures and temperatures than the following ones [8,9,25]. For the second and the following layers, the lamination parameters do not change.

In the present study, we report on the fabrication of a novel microfluidic system that features three-level DFR processing in combination with thin-film metal deposition for advanced electrical impedance spectroscopy (EIS) to characterize hydrodynamically trapped single objects. The combination of DFR multi-level processing with embedded, vertically arranged electrodes around an aperture for four-point measurements ensures high impedance signals. For proof-of-concept, impedance measurements were carried out on polystyrene and ZrO_2_ spheres with different diameters, which show the highest impedance signal sensitivities at approximately 2 kHz.

## 2. Materials and Methods

### 2.1. Materials

First, 25 µm thick SUEX K25 DFR and mr-Dev600 developer were purchased from micro resist technology GmbH (Berlin, Germany); AZ10XT photoresist, AZ40XT-11D photoresist and the chemicals for photolithography from MicroChemicals GmbH (Ulm, Germany); gold electroplating solution NB SEMIPLATE AU 100 TL from NB Technologies GmbH (Bremen, Germany); PDMS (Sylgard 184 kit) from Farnell GmbH (Aschheim, Germany); phosphate-buffered saline (PBS) tablets P4417, acetone, isopropanol, hydrochloric acid, nitric acid, hydrogen peroxide solution and ammonium hydroxide solution from Sigma-Aldrich Chemie GmbH (Taufkirchen, Germany); PS-FluoRot-60 and PS-FluoRot-80 polystyrene (PS) spheres (60 µm PS sphere and 80 µm PS sphere) from microParticles GmbH (Berlin, Germany) and SiLibeads Typ ZY-P Pharma (90 µm ZrO_2_ sphere) from Sigmund Lindner GmbH (Warmensteinach, Germany).

### 2.2. System Concept

**Figure 1:**
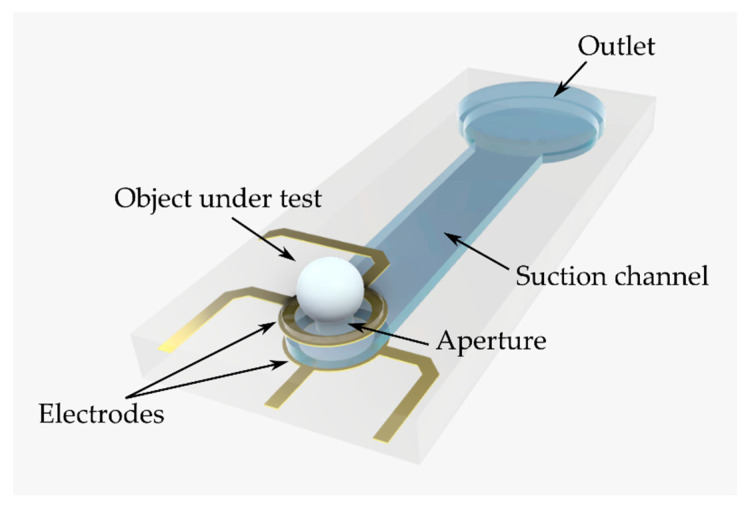
3D schematic depiction of the microfluidic chip. An object is trapped and fixed at the aperture through the microfluidic suction channel. The object is characterized by electrical impedance spectroscopy using two vertically adjusted, ring-shaped microelectrodes around the aperture. Drawing not to scale.

The design of the microfluidic chip for the characterization of trapped objects by EIS is schematically illustrated in Figure 1. The chip consists of a microfluidic channel and thin-film gold microelectrodes. At the inlet, an aperture serves as a hydrodynamic trap for objects. At the rim of the aperture, two vertically adjusted, ring-shaped electrodes are embedded. Each electrode is connected with two wirings to enable 4-point electrical impedance measurements. Through the outlet of the microfluidic channel, trapping and fixation of objects can be achieved by applying negative pressure. For exchange, objects can be released from the aperture by applying positive pressure to the outlet.

### 2.3. Equivalent Electric Circuits

**Figure 2:**
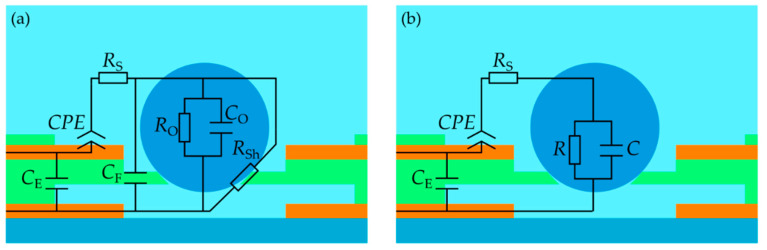
Schematic cross-section of the measurement unit with the equivalent electric circuit (EEC) on the object under test. (**a**) Full EEC. *CPE*: constant phase element of electrode/electrolyte interface, *R*_S_: solution resistance, R_O_: resistance of the object under test, *C*_O_: capacitance of the object under test, *R*_Sh_: shunt resistance near the rim of the aperture, *C*_F_: capacitance of the thin-film around the aperture, *C*_E_: crosstalk capacitance of vertically arranged electrodes at high frequencies. (**b**) Simplified EEC for fitting the electrical impedance spectroscopy (EIS) spectra. Drawing not to scale.

Experientially obtained impedance data are typically simulated by equivalent electric circuits (EEC) [26,27,28,29,30]. The circuit is proposed in Figure 2a. The electrode/electrolyte interface can be modeled by a modified Randles model [31]. Since there are no electrochemical reactions expected at the interface, charge transfer does not take place and therefore no charge transfer resistance needs to be considered. The electric double layer at the interface is described by a constant phase element *CPE*, which represents a non-ideal capacitive electrode [32,33]. Its impedance is calculated by [34,35]

(1) $$Z_{\mathrm{CPE}}=\frac{1}{CPE{\left(j\omega \right)}^{n}},$$

where *ω* represents the radiant frequency, whereas *n* (0 ≤ *n* ≤ 1) describes for *n* = 1 an ideal capacitance and for *n* = 0 a pure resistance. *R*_S_ is associated with the resistance of the electrolyte solution, whereas *C*_E_ represents the crosstalk capacitance of vertically arranged electrodes and the wirings at high frequencies. An object under test can be treated as a resistance *R*_O_ and a capacitance *C*_O_ in parallel. In the event that an object is trapped and the aperture is not in close contact with the rim of the aperture, the gap can be described by a shunt resistance

(2) $$R_{\mathrm{Sh}}=\frac{1}{\Phi }R_{\mathrm{Sh}0},$$

where *Φ* (0 ≤ *Φ* ≤ 1) represents the leakage rate. For *Φ* = 0, no leakage exists due to the completely sealed aperture. For *Φ* = 1, the aperture is fully open, which is represented by the initial shunt resistance *R*_Sh0_. *C*_F_ describes the capacitance of the thin film of DFR between the aperture and the electrodes. Since *R*_O_, *C*_O_, *R*_Sh_ and *C*_F_ are arranged in parallel, these components can be summarized as a resistance *R* and a capacitance *C*, as depicted in Figure 2b. Therefore, the resistance is calculated by

(3) $$R=\frac{R_{O}R_{\mathrm{Sh}}}{R_{O}+R_{\mathrm{Sh}}},$$

combined with Equation (2), this equation can be written as

(4) $$R=\frac{\frac{1}{\Phi }R_{\mathrm{Sh}0}R_{O}}{\frac{1}{\Phi }R_{\mathrm{Sh}0}+R_{O}},$$

the capacitance of *C* can be expressed as

(5) $$C=C_{O}+C_{F},$$

the simplified EEC is used to perform the EIS fitting and the impedance of the EEC in can be calculated by

(6) $$Z_{\mathrm{EEC}}=\frac{1}{\frac{1}{1/R+j\omega R}+R_{S}+\frac{1}{CPE{\left(j\omega \right)}^{n}}}+j\omega C_{E}.$$

### 2.4. Microfluidic Chip Fabrication

**Figure 3:**
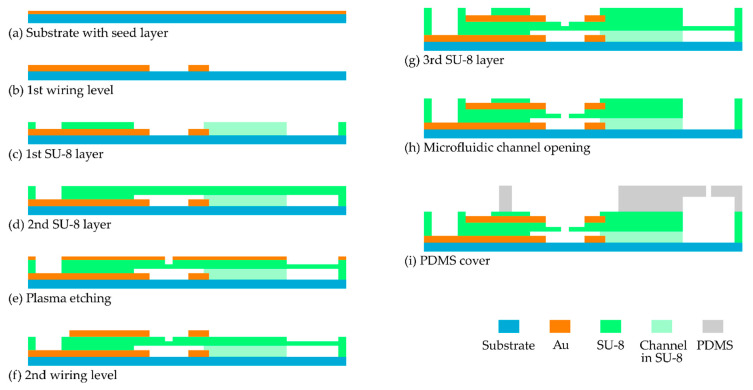
Process flow for the fabrication of the microfluidic chip for EIS measurement of trapped objects under test. (**a**) Deposition of a Ti/Au seed layer. (**b**) Lithography, Au electroplating of 1st wiring level and resist and seed layer strip. (**c**) Lamination and patterning of the 1st SU-8 dry film photoresist (DFR). (**d**) Lamination and lithographic patterning of the 2nd SU-8 DFR. (**e**) Deposition of a Ti/Au seed layer. After the first sub-lithography, the Ti/Au layer is patterned and the 2nd SU-8 DFR is partly dry-etched. (**f**) After the second sub-lithography step, electroplating of Au is carried out for the 2nd wiring level. Etching of seed layer. (**g**) Lamination and patterning of the 3rd SU-8 DFR as a passivation layer. (**h**) Opening of the microchannel. (**i**) Bonding a poly(dimethylsiloxane) (PDMS) cover on the microfluidic chip. Drawings not to scale.

The microfluidic chip was developed using MEMS technologies including multilayer DFR lamination. The fabrication process is depicted schematically in Figure 3.

Microfluidic chips were fabricated on 4-inch glass substrates. First, a seed layer (10 nm titanium (Ti)/100 nm gold (Au)) was deposited using an e-beam evaporator Leybold A700 QE (Leybold GmbH, Cologne, Germany) (Figure 3a). AZ10XT photoresist was coated on the substrate using a spin coater Süss MicroTec RC8 (Süss MicroTec, Garching, Germany) and patterned with a mask aligner Süss MicroTec MA6 (Süss MicroTec, Garching, Germany). Then, 2 µm thick gold (Au) was electroplated (Figure 3b). After surface activation by oxygen plasma, the 1st SUEX DFR sheet was laminated using a Royal Sovereign RSL 382 S roll laminator (Royal Sovereign International Inc., Rockleigh, NJ, USA), whose up and down rollers were set to a temperature of 95 °C and 85 °C, respectively, with a laminate speed of 1 cm/s and a pressure level of 3.5. This first DFR layer, which serves as a passivation layer for the 1st wiring level and as the sidewalls of microfluidic channels, was lithographically patterned with i-line exposure at a dose of 1440 mJ/cm^2^. After a post-exposure bake at 85 °C for 45 min, the wafer was cooled down to 21 °C in 3 h and developed in mr-Dev 600 for 15 min in 2 steps. The wafer was afterward ramped up to 200 °C in 1 h, hard-baked for 2 h and ramped down back to 21 °C in 4 h to ensure the durability of the laminated DFR layer (Figure 3c). Then, a 2nd SUEX DFR sheet was laminated with a pressure level of 3 and lower roller temperatures, i.e., 85 °C for the upper and 75 °C for the lower roller. Afterward, the second layer was lithographically patterned and processed as described for the 1st SUEX DRF sheet. The layer gives access to the contact pads and additionally covers the microfluidic channels (Figure 3d). A second seed layer (10 nm Ti/100 nm Au) was deposited. Two sub-lithography steps were carried out: after the first one, the metal film was structured wet-chemically. Subsequently, the 2nd DFR layer was thinned down to approximately 10 µm by O_2_/SF_6_ plasma etching process using a plasma etcher Tegal 901 (Tegal Corporation, Petaluma, CA, USA). For a fast etching process, the flowrates of O_2_ and SF_6_ were adjusted to 50 sccm and 2 sccm, respectively [36,37] (Figure 3e). After the second sub-lithography step, the top Au electrodes and wirings were defined and the Au layer was thickened to 2 µm using electroplating (Figure 3f). Afterward, a 3rd SUEX DFR sheet was laminated, lithographically patterned and processed as described for the 2nd SUEX DFR sheet, which gives access to the electrodes and the microchannels (Figure 3g). Subsequently, the glass wafer was diced into chips. A dry-etching process in O_2_/SF_6_ plasma removed the rest of the thinned 2nd SU-8 layer. The unveiled aperture provides access to the microchannel as an inlet (Figure 3h). In the last step, a PDMS cover was bonded to the chip. The cover was made by standard replica molding (Figure 3i).

## 3. Results

### 3.1. Measurement Setup

**Figure 4:**
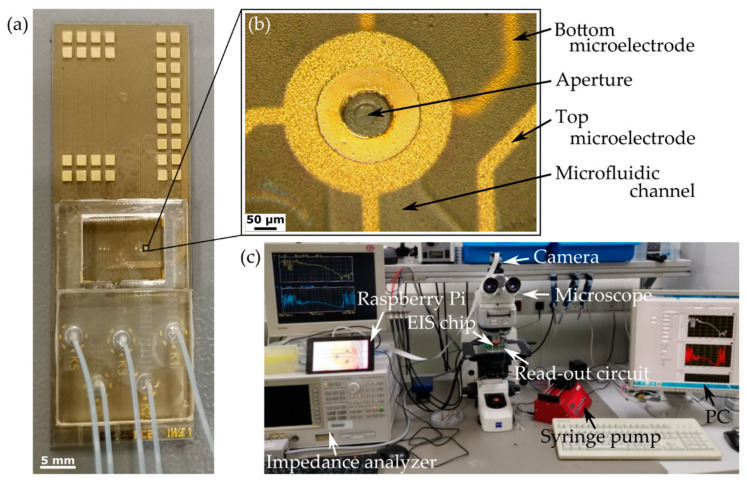
Photos of measurement setup. (**a**) Top view of fabricated microfluidic chip: upper part: contact pads, middle part: chamber with EIS measurement units, lower part: interposer of PDMS with Teflon tubes. (**b**) Top view of an EIS measurement unit with ring-shaped microelectrode and central aperture with a diameter of around 51 µm. (**c**) The complete measurement setup.

Figure 4a shows the fabricated microfluidic chip. The chip has a footprint of 25 mm × 68 mm. The upper area is not covered with PDMS, but provides 36 electric contact pads for the electrodes of nine measurement units. The chip’s central area is surrounded by a PDMS frame. Inside the frame, the nine measurement units are located. The lower part of the chips is covered with an interposer made of PDMS to connect the outlets with Teflon tubes (polytetrafluoroethylene (PTFE), 0.3 mm ID × 0.7 mm OD). The tubes are subsequently connected to the syringes, which are driven by syringe pumps (AL-1000, World Precision Instruments, Friedberg, Germany). Figure 4b shows the top view to one of the nine EIS measurement units. The top and bottom ring-shaped microelectrodes have the same dimensions (360 µm outer diameter, 130 µm inner diameter, 2 µm thick). The inner part of the ring-shaped electrodes with a width of 35 µm is exposed, while the rest is passivated by the SUEX DFR layer. The aperture has a diameter of around 51 µm. The surface of the top SUEX DFR layer is rough, which is caused by the ion bombardment during the aperture opening dry-etching process. The complete measurement setup is shown in Figure 4c. The chip is electrically connected to a 4294A precision impedance analyzer (Keysight, Santa Rosa, CA, USA) via a custom-made read-out circuit. Continuous EIS measurement and data acquisition are performed using a custom-made LabView program (National Instruments Inc., Austin, TX, USA). The measurements are monitored by a Raspberry Pi with a camera (Raspberry Pi (Trading) Limited, Cambridge, UK) through a light microscope ZEISS Axio Imager (Carl Zeiss AG, Oberkochen, Germany).

### 3.2. Electric Impedance Spectroscopy on Single Polystyrene Spheres

**Figure 5:**
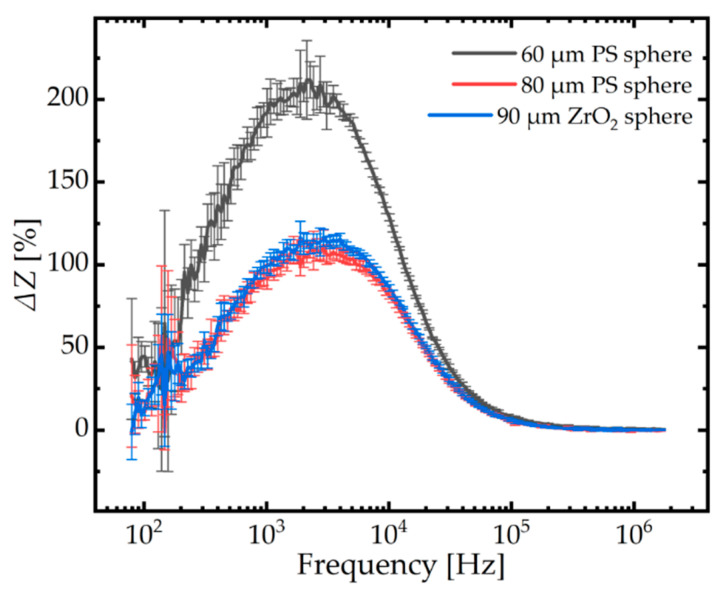
Normalized electric impedance change $\Delta Z=Z_{m}/Z_{0}-1$ spectra from 80 Hz to 2 MHz of polystyrene and ZrO_2_ spheres. *Z*_m_ and *Z*_0_ refer to the magnitude of the impedance of a measurement unit filled with a sphere and a measurement of an empty unit in 1 mM phosphate-buffered saline (PBS), respectively. Error bars of standard deviation are plotted.

The chamber was filled with 1 mM PBS solution. Single polystyrene or ZrO_2_ spheres were transferred in the vicinity of an EIS measurement unit with the help of a pipette (Denu Tips 125 µm and EZ-Grip, GYNEMED GmbH & Co. KG, Lensahn, Germany) using polyamide tips with an inner diameter of 125 µm. The EIS measurements were carried out between 80 Hz and 2 MHz with a voltage amplitude of 5 mV. Figure 5 shows the normalized impedance $\Delta Z=\frac{Z_{m}}{Z_{0}}-1$ with respect to the applied frequencies. *Z*_m_ and *Z*_0_ refer to the magnitude of the averaged impedance of a measurement unit filled with a single sphere and that of the empty unit in 1 mM PBS, respectively. Both types of spheres are electrically sensitive between 80 Hz and 300 kHz. The normalized impedance shows maximum values at 2 kHz. The PS sphere of 60 µm in diameter shows the highest normalized impedance value of around 210% compared to the one found for the PS sphere of 80 µm in diameter and the ZrO_2_ sphere of 90 µm in diameter (around 120%). No significant difference could be observed between the normalized impedance values of the two larger spheres.

**Figure 6:**
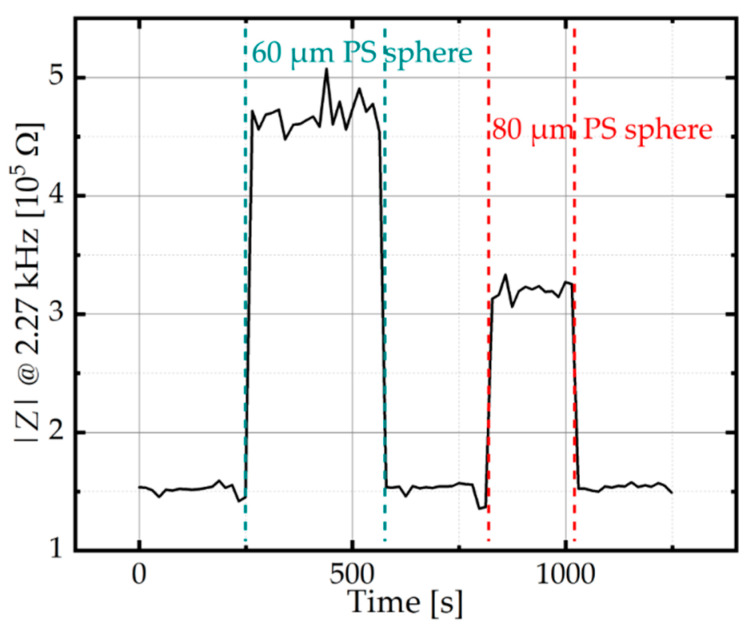
Time dependency of the impedance magnitude at 2.27 kHz during trapping and release of single polystyrene (PS) spheres (60 µm and 80 µm in diameter) in 1 mM PBS, respectively.

The electric impedance spectra during the trapping and releasing procedures were continuously measured and recorded approximately every 15 s. Figure 6 shows the magnitude of electric impedance at 2.27 kHz over time during the trapping and release procedure. During trapping of PS spheres that were 60 µm and 80 µm in diameter, the impedance magnitude increased. Trapping of the larger sphere resulted in a higher increase in the impedance magnitude. No signal drifts were observed.

### 3.3. EIS Spectra and Fitting with Equivalent Electric Circuits

**Figure 7:**
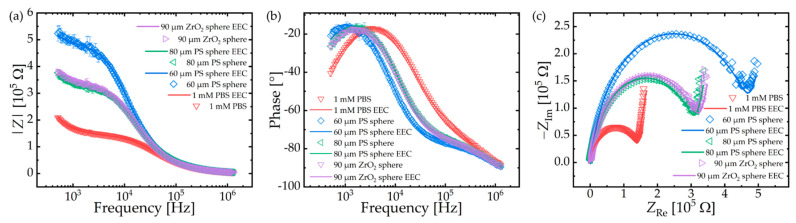
Electrical impedance spectra of 1 mM PBS, 90 µm diameter ZrO_2_ sphere, 60 µm and 80 µm diameter polystyrene spheres, respectively. Measurements are represented in points with standard deviation as error bars. Curves represent the fitting by the equivalent electric circuit model. (**a**,**b**) Bode diagrams and (**c**) Nyquist plot.

Figure 7 depicts Bode diagrams (Figure 7a,b) as well as Nyquist plots (Figure 7c) of the EIS measurements in PBS, carried out with the two PS spheres of different diameters and with the ZrO_2_ sphere, respectively.

The EEC model proposed in Figure 2b was used to fit the impedance spectra. For data fitting, the Powell algorithm (300 iterations) and the open-source software “EIS Spectrum Analyser” (http://www.abc.chemistry.bsu.by/vi/analyser/, accessed on 1 February 2021) were used, which was developed by Ragoisha and Bondarenko [38]. The parameter of each equivalent component in the EEC model was simulated and the relative errors were given by the software. First, the impedance spectrum of a measurement unit only filled with 1 mM PBS was taken and fitted. The *CPE* depends on the ion concentration of the electrolyte, whereas *C*_E_ depends on crosstalk between the electrodes and the whole circuitry of the measurement setup at high frequencies. Therefore, these two parameters (*CPE*: 8.22 × 10^−9^ F (3.32%) with *n* = 0.84 (0.43%); *C*_E_: 2.83 × 10^−11^ F (1.46%)) were fixed for the fittings of the impedance spectra with spheres. The values of the other circuit elements are listed in Table 1. The fitted curves are presented in Figure 7 as solid lines.

As shown in Figure 7, the capture of a PS sphere with a diameter of 60 µm caused an impedance increase in the lower-frequency range and a negative phase shift of around 2 kHz. Larger spheres show lower impedance values compared to the smaller ones because the filling of the aperture is more pronounced for smaller objects.

## 4. Discussion

The aim of this work was the development of a three-level DFR lamination process of SUEX DRF with embedded microelectrodes for electrical impedance spectroscopy. Using the novel fabrication process, the fabricated microfluidic system enables hydrodynamic trapping and the fixation of single objects to the microelectrodes. The presented process shows the unique capability of using DFRs. Compared to liquid thick-film processing or other 3D-capable microfabrication technologies, such as ultraprecision micromachining [39,40], wet etching [41] or laser micromachining [42], the processing with DRF is easy to carry out and substantially saves process time. Previously, we developed a six-level DRF process for complex microfluidic devices, but without the integration of microelectrodes [25].

Using DRFs, the challenge in our work here was the integration of vertically adjusted ring-shaped microelectrodes around the inlet aperture of the suction channel. The two electrodes were designed as intermediates between the DFR levels (see Figure 3) and fabricated using e-beam evaporation and electroplating of gold. The lamination processes of the first DFR level were optimized to achieve layer bonding on a glass substrate without leakage around the 2 µm thick gold wires, which connect the microelectrodes. O_2_ plasma activation of the glass substrate is essential to provide strong bonding between the glass substrate and the SUEX DFR sheet. It has to be noted that microelectrodes embedded in a SU-8-based microfluidic device were already developed in [43], but sophisticated liquid SU-8 processing was used.

Another challenge was the fabrication of the aperture in combination with the suction channel. As shown in Figure 3, the process sequences were arranged in such a way that filling of the microchannels during the wet-chemical development step of the lithography process was avoided. Therefore, for patterning the tiny aperture, the SU-8 was first structured only partly. Later, in one of the following process steps, the aperture was released. For the two-step structuring, a dry-etching process in O_2_/SF_6_ plasma was used. The addition of SF_6_ to O_2_ increases the etching rate of SU-8. Only with this two-step approach, the aperture was successfully manufactured without filling the microfluidic channel underneath with liquids during processing.

For the aperture, the opening diameter on the mask was designed to be 10 µm, which is the resolution limit of the used foil masks. After etching through the 25 µm thick SUEX DFR, the diameter was enlarged to 51 µm, as shown in Figure 4b. The plasma etching process was nearly isotropic. By optimization of the etching parameters in the future, the process could become more anisotropic, in order to further reduce the size of the aperture. In addition, chromium masks for higher pattern resolution can be used to avoid a non-circular aperture shape as can be observed in Figure 4b.

To avoid sagging of the lid layer of the microfluidic channels, the pressure and the lamination temperature of the second and third DFR layers were reduced. As already noted by several authors [8,9,25] and conformed here, the process parameters of DFR multi-level lamination need only to be adjusted for the second level, whereas, for all further levels, the process parameters of the second level can be used. It has to be noted that a few micro-cavities were formed between the laminated DFR layers, but no influence on the passivation of wirings was noticed.

Figure 4a shows the processed chip. The chip area is divided into three segments. On the upper part, pads are arranged periodically to enable easy contact with pogo-pins. The middle and lower segment are covered with PDMS. In the middle segment, the nine EIS measurement units are arranged within a PDMS frame. A detailed top view of one unit is shown in Figure 4b, with its ring-shaped top electrode, the aperture in the center and the four wires for connecting the two electrodes for four-point EIS measurements. The microchannel is expanding to the lower right side. The bonding of the PDMS cover to the SU-8 was ensured mechanically with a clip.

For the experiments, the objects under test were placed in the vicinity of the aperture via a pipette. The objects can be easily trapped and fixed to the aperture by sucking fluid through the aperture into the microfluidic channel with a syringe. Compared to overall microchannel-based setups, this simple placement handling has the advantage that the object under test must not flow through long inlet tubes with high probabilities of sticking to the walls and getting caught in corners, especially at the right angles of the inlet channels. Furthermore, the sizes of objects under test are not restricted to the diameter of the inlet tubes and channels.

For the proof-of-concept, polystyrene spheres with different diameters and a ZrO_2_ sphere were characterized by EIS. The maximum values of the relative impedance magnitudes were observed at 2 kHz, as shown in Figure 5. The high maximum values were obtained due to the arrangement of electrodes and the aperture (as depicted in Figure 2), in which all electrical field lines are concentrated by the aperture. This guarantees a highly sensitive impedance signal. The low frequency range at which the spheres are electrical sensitive enables the use of a cheap and portable EIS spectrometer instead of the used expensive and bulky analyzer with its high-frequency range.

To investigate the properties of the measurement unit in detail, Bode diagrams and Nyquist plots of empty and filled measurement units were compiled and are shown in Figure 7. In addition, the graphs, which were obtained by simulating the electrical equivalent circuit proposed in Figure 2b, were also plotted. The experimental results fit excellent with the simulated graphs. The values of the circuit elements are listed in Table 1. From the impedance spectrum of PBS in an empty measurement unit, we obtained the constant phase element *CPE* of the electrode/electrolyte interface as well as the crosstalk capacitance *C*_E_ at high frequencies.

The solution resistance *R*_Sol_ increased after the capture of a PS sphere at the aperture. This increase is dependent on the size of the spheres (see Table 1). As shown in Figure 4b, the aperture is not ideally circularly shaped, which prevents close contact of the sphere with the aperture rim. This has a significant influence on the resistance *R* (see Figure 2 and Table 1). The resistivity of polystyrene is 1.5 × 10^13^ Ω·m and the resistivity of 1 mM PBS is estimated to be around 5.8 × 10^2^ Ω·m [44,45]. For *R*_O_ >> *R*_Sh0_, Equation (4) can be simplified to

(7) $$R=\frac{1}{\Phi }R_{\mathrm{Sh}0},$$

for Φ=1, the aperture is completely exposed to PBS. This corresponds to the EIS spectrum with pure 1 mM PBS. Therefore, the shunt resistance *R*_Sh0_ equals resistance *R* of a value of 6.9 × 10^4^ Ω in Table 1. With regard to the resistances of the spheres in Table 1, the leakage rates of the 60 µm PS sphere, 80 µm PS sphere and 90 µm ZrO_2_ sphere are calculated as 18.7%, 31.1% and 29.5%, respectively, by applying Equation (7). The aperture is more sealed by the 60 µm PS sphere; therefore, the impedance values shown in the Bode diagrams and the Nyquist plots are higher. As discussed above, the leakage can be reduced after process optimization by using chromium masks as well as an anisotropic reactive etching process of SUEX for creating the ideally circular aperture shape.

On the other hand, no dependency on the sphere material is indicated by the observed values of R, since the resistivity of the dielectric sphere is typically much higher than the resistivity of the surrounding media. At the low-frequency range, where the spheres show an impedance response, the conduction current dominates, so that substantially all of the current is shunted in the medium between the probing microelectrodes.

As shown in Table 1, the capacity *C* decreases with the capture of a sphere but does not change significantly for spheres of different sizes or different materials, which means that, according to Equation (5), the equivalent capacity *C*_O_ of the sphere is too small to influence the combined capacity C, although the dielectric constant shows values between 2.4 and 3 for PS and between 10 and 23 for ZrO_2_.

## 5. Conclusions

We presented a process technology of a three-level DFR lamination process of SUEX with embedded microelectrodes. The novel microfluidic chip design enables electrical impedance spectroscopy of single trapped objects. Two ring-shaped electrodes were vertically arranged around the aperture of a microfluidic suction channel. By applying pressure at the suction channel, the chip offers easy handling, trapping, fixation and release of single objects under test. The chip was fabricated by laminating three layers of SUEX dry film resist in combination with lithography, electroplating and dry-etching processes. The fabricated microfluidic EIS chip was characterized using PBS and two types of microspheres. Electrical impedance spectra revealed that the highest relative impedance signals were observed at a frequency of around 2 kHz. The proposed setup may be used to determine non-destructively and label-free single micro-objects by EIS.

## Figures and Tables

**Table 1:** Values of variable parameters of the equivalent electric circuit model proposed in Figure 2b of different spheres as well as of 1 mM PBS simulated by Powell algorithm using the software “EIS Spectrum Analyser”. Relative errors are given in brackets.

Parameter (Rel. Error).	*R*_Sol_ [Ω]	*R* [Ω]	*C* [F]
1 mM PBS	6.95 × 10^4^ (1.67%)	0.69 × 10^5^ (1.85%)	4.78 × 10^−11^ (2.18%)
60 µm sphere	10.5 × 10^4^ (2.32%)	3.69 × 10^5^ (1.39%)	1.86 × 10^−11^ (3.79%)
80 µm sphere	9.09 × 10^4^ (2.91%)	2.22 × 10^5^ (1.63%)	2.30 × 10^−11^ (3.21%)
90 µm ZrO_2_ sphere	9.08 × 10^4^ (2.73%)	2.34 × 10^5^ (1.53%)	2.22 × 10^−11^ (4.79%)

## Data Availability

The data presented in this study are available on request from the corresponding author.
